# Patients’ experiences of adverse symptoms, emotions, and coping strategies in connection to treatment of head and neck cancer - an interview study

**DOI:** 10.1186/s12903-023-03366-4

**Published:** 2023-09-05

**Authors:** Ellie Saghafi, Charlotte Andrén Andås, Jenny Bernson, Göran Kjeller

**Affiliations:** 1 Department of Oral and Maxillofacial Surgery, Institute of Odontology, Sahlgrenska Academy, University of Gothenburg, Medicinaregatan 12A, Gothenburg, 413 90 Sweden; 2 Department of Orofacial Pain, Region Västra Götaland, Institute of Odontology, Sahlgrenska Academy, University of Gothenburg, Gothenburg, Sweden; 3 Department of Cariology, Institute of Odontology, Sahlgrenska Academy, University of Gothenburg, Gothenburg, Sweden

**Keywords:** Head and neck neoplasms, Radiotherapy, Symptoms, Qualitative research

## Abstract

**Purpose:**

This study aimed to increase the understanding of emotions and coping strategies used by head and neck cancer patients before cancer treatment, and to explore their emotions and coping strategies in relation to symptoms and side effects after treatment. Furthermore, we aimed to investigate the patients’ perceptions of received treatment and support.

**Methods:**

Semi-structured in-depth interviews were conducted with 10 patients who had been treated for head and neck cancer, which included radiotherapy, at the Department of Oncology and the Department of Oral and Maxillofacial Surgery at Sahlgrenska University Hospital in Gothenburg. The interviews were analyzed in accordance with the method for Qualitative Content Analysis.

**Results:**

The result picture revealed three head themes. The first theme “*Management of simultaneously influencing mind-sets before cancer treatment*” described the patients experiences of feeling *“Scared and worried,” “Lonely and disappointed,”* and *“Relieved and confident”*, and how they tried to handle the diagnosis and preparations for treatment by “*Applying a positive mind-set”, “Searching for support,”* and *“Trusting the healthcare system*”. The second theme *“Experiences of becoming a pale shadow of oneself*”, illustrated experiences of affecting post-treatment symptoms and side effects. To which, the last theme “*Handling contextual influencing experiences after cancer treatment*” displayed post*-*treatment emotions of being *“Shocked and disappointed”* and *“Concerned and unsupported”* but also *“Grateful and forward-thinking”*, where strategies such as *“Appreciating Life”, “Networking socially,”* and *“Adapting to the new life”* were used.

**Conclusions:**

The results indicated the need for a more patient-centered care approach, with clearer structures and improved individual support both before and after treatment and in connection to rehabilitation. Patients’ cognitive changes after cancer treatment should be considered in the aftercare, which should also include adaptation to situation and strengthening of patients’ self-management as a goal.

## Background

Head-neck tumors are a collective name for a heterogeneous group of malignancies that potentially have serious effects on many basal functions [1]. Head and neck cancer (HNC) may occur at several different anatomical locations such as the oral cavity, the pharyngeal tract, the nasal cavity, the sinuses, or the larynx [2]. The incidence of HNC is reported to be steadily increasing worldwide and on a global level, it constitutes the sixth most common type of cancer [3]. However, there are significant anatomical and geographical variations. Of the about 68,000 new cancer cases diagnosed in Sweden in 2020, approximately 1,500 were HNC. Most sufferers are over 65 years of age and HNC is most prevalent in older males [4], however, the human papillomavirus (HPV) and HPV-related oro-pharyngeal cancer are reported to be increasing in young people [5]. The main cause of HNC is considered to be lifestyle factors such as tobacco and alcohol exposure. Many patients can be cured, but the outcome after treatment varies greatly depending on the patient’s general health status as well as the type of cancer, anatomical location, and tumor stage [6].

The therapies available for head-neck tumors include radiotherapy, chemotherapy, and surgery, often in combination [7, 8]. Several basic functions can be affected by both the tumor and the subsequent treatment. Chewing, swallowing, breathing, smelling, and tasting as well as the patient’s physical appearance may be affected to varying degrees.

Pain and oral dysfunctions included in the umbrella term, “temporomandibular dysfunction (TMD)”, and resulting in impaired quality of life are frequently reported among patients experiencing post-treatment symptoms. Survivors of HNC may experience additional consequences after radiotherapy, which includes dysphagia, xerostomia, impaired or loss of speech, fatigue, and cognitive impairment [9–11]. Further a restricted mouth opening is a consequence which has been shown to significantly affect a patient’s health-related quality of life [12]. TMD symptoms seem to be more prevalent in patients with psychosomatic symptoms, where perceived stress and previous life events also predict TMD incidence [13, 14].

The relationship between cancer, pain, and mental well-being is complex. Suicide is more common among patients diagnosed with HNC in comparison to the general population [15].

HNC patients’ lived experiences post-treatment and their emotional concerns have been described in a recent qualitative study [16], where the participants perceived a general gratitude for life and positive mentality, despite the restrictions on daily living, social eating, and financial concerns.

To manage the perceived post treatment difficulties the patients, use different coping strategies.

Coping has been defined as cognitive and behavioral efforts to manage internal and external stressful demands [17]. Coping is considered to be dependent on personal apprehension of the situation and on a wide range of individual reactions. Emotions, motivation, attention, volition, cognitions, and communication are examples of such reactions. Physiological functioning and many aspects of social relationships and cultural systems are also said to contribute to the use of coping strategies [17]. Post-treatment coping strategies in HNC patients were also described by Dunne et al. [18]. These included adapting to a new normal life, and increased involvement in cancer support and faith groups. The most common self-management strategies used by HNC patients, which included strategies such as self-sustaining, self-motivating and proactive problem-solving was presented [18].

It has been suggested that psychological interventions for patients with HNC and their caregivers should be delivered early after diagnosis, presenting honest and factual information about the disease and coping strategies, in face-to-face sessions [19]. Previous qualitative studies have also explored the function of this patient group in terms of food intake and pain [20]. Other studies investigating various physical, social, and emotional impacts of HNC on survivors have captured the association to a perceived quality of life by quantitative measurements [21–23].Further qualitative studies have shown that HNC survivors continue to have unmet needs in regards to supportive care services for adapting to life after treatment and for managing long-term health consequences of their disease [16, 24].

In Sweden, if HNC is suspected, the diagnostics are carried out in accordance with a standardized care procedure [25]. Use of the procedure aims to reduce time between suspicion of malignancy and final diagnosis through a strict and ordered sequence of investigations. Often there is a combination of healthcare modalities involved in the treatment of patients with HNC, dependent on the location of the tumor and the treatment needs. Ear, nose, and throat (ENT) doctors, maxillofacial surgeons, plastic surgeons, oncologists, psychologists, dieticians, general dentists, and orofacial pain specialists are some of the occupational groups that can be involved in both the treatment and rehabilitation of HNC patients. The involvement of different healthcare professions can sometimes make the collaboration difficult since the location of the facilities and the healthcare management varies depending on the type of profession. It is of importance to restitute the functions as early as possible in the aftermath of HNC treatment. A well-planned rehabilitation is therefore necessary. This, however, frequently implies additional care visits, for instance to prosthodontists or anaplastologists. It is of interest to evaluate how the HNC patients perceive the cancer care in Sweden. This study follows the informant’s path from the emotions and handling of the diagnosis to the long-term coping strategies post-treatment, which can give us a better understanding of the patients’ experience of the “whole picture”. This may provide crucial information regarding where in the process the healthcare system can, and should, provide better support or care. As with many other conditions, the development of new preventive and supportive treatment methods, e.g., through identification of possible pre-treatment factors that can facilitate preventive interventions and lead to faster, more effective treatment and rehabilitation, is desired for this patient group.

### Aim

The aim of this study was to gain a better understanding of the emotions and coping strategies used by HNC patients before cancer treatment. Further, to explore patients’ experiences of emotions and coping strategies used in relation to adverse symptoms and problematic side effects after treatment and finally, to investigate the patients’ perceptions of received treatment and support.

## Materials and methods

A qualitative methodology approach was chosen to obtain a deeper understanding and knowledge about Swedish patients’ experiences and management of postoperative symptoms after treatment of HNC. The study data was collected from semi-structured in-depth interviews with patients who earlier had undergone treatment for HNC. The choice fell on qualitative content analysis [26, 27], a well-described and a frequently used method, not at least in healthcare. The method is systematic and designed to explore and interpret both the manifest and latent meaning in texts and to be able to draw conclusions about the content of text data at different levels of abstraction, where a deeper understanding and increased knowledge of the participants experiences are desired. However, the basis for qualitative content analysis is the assumption that acquired information can always be interpreted in different ways and thus include several different meanings. Content analysis is best understood as a broad family of techniques. Effective researchers choose the techniques that best help them answer their question [26, 27].

The analysis of the study´s data was carried out with the addition of a quality-enhancing collection and analysis step. In order to increase the credibility of the recruitment of informants and the collection of data, the constant comparative analysis of the data was implemented, i.e. the analysis and possible co-analysis with previous interviews took place immediately after the interviews had been carried out. Only then was the next informant summoned. This procedure enabled strategic and theoretical selection of informants, which gave rise to increased variety and quality of data, as well as the opportunity to perceive when data saturation occurs, that is, when no new data appears that contributes to changing the result picture [28]. Although the constant comparative analysis is a characteristic of Grounded Theory [29], it can be used in other qualitative research with the collection of data through successive collections, e.g. interviews. The concept of saturation is also found in most guidelines for various qualitative research methods. Additional characteristics are that the analysis is carried out within the context or the contexts the question is related to, which is important for any findings that emerge [28].

### Recruitment of the informants

The informants were patients who were registered and treated for HNC in the region of Västra Götaland, Sweden. The inclusion criteria were adult patients (> 18 years), diagnosed and treated for HNC at the Department of Oncology and the Department of Oral and Maxillofacial Surgery at Sahlgrenska University Hospital in Gothenburg. Cancer treatment must have been completed more than two years prior to study start and have included radiotherapy. Exclusion criteria were difficulties speaking and understanding the Swedish language and suffering from severe physical or mental illness with loss of own autonomy.

Patients were identified using ICD-10 (C00.X – C14.X) codes for HNC in their medical records. Upon invitation to the study, potential participants received both oral and written information about the purpose of the study and its confidentiality. All participants were first contacted by telephone. All respondents asked were willing to participate and gave both oral and written informed consent.

At the beginning of the study a convenient selection of informants was applied, in order to switch to a more strategic selection towards the end, this for maximum variation and quality in data.

A total of ten informants participated in the study, five men and five women, 42–72 years of age. They were all residents in Sweden but three were born in other countries (China, Denmark, and Somalia). All participants had received both chemotherapy and radiotherapy to the head and neck region. Six of the informants had also undergone surgery. Patient characteristics are described in Table 1.

**Table 1:** Patient characteristics

Participant	Gender	Age	Marital status	Site of primary tumor	Treatment	Years passed since last treatment
P1	M	64	Married	Oral cavity	Chemotherapy, radiation and surgery	3
P2	F	51	Divorced	Epipharynx	Chemotherapy and radiation	5
P3	M	50	Married	Oral cavity	Chemotherapy, radiation and surgery	6
P4	F	42	Married	Nasopharynx	Chemotherapy, radiation and surgery	5
P5	M	72	Single	Oral cavity	Chemotherapy and radiation	6
P6	F	43	Partner	Oral cavity	Chemotherapy, radiation and surgery	5
P7	F	69	Divorced	Epipharynx	Chemotherapy and radiation	3
P8	F	59	Partner	Oral cavity	Chemotherapy, radiation and surgery	4
P9	M	70	Married	Nasopharynx	Chemotherapy and radiation	7
P10	M	53	Married	Oral cavity	Chemotherapy, radiation and surgery	5

### Data collection

An interview guide was developed based on a literature review connected to the study background and further modified by the research group after three pilot tests, before the first interview. The guide included topics addressing patients’ experiences of post-treatment symptoms and accompanying difficulties, questions about emotions, ways to think and to handle the situation, as well as experiences of the treatment and support given in dental and medical care. The open, semi-structured interview format enabled an in-depth exploration of various data-themes through appropriate investigation and follow-up questions, whereby the interview guide had possibilities to change over time. Important in the collection process of data, was that the researchers had a good understanding of the research process and research area. This means a possession of theoretical sensitivity and specificity with such a pre-understanding that the data is perceived as extra important for the work can be found [29].

A total of ten interviews were conducted by three researchers and either held and audio-recorded in a suitable room at the Department of Odontology, University of Gothenburg, or in the informants’ home, when interviews via digital technology (Microsoft® Teams) were also offered. No relationship was established between interviewer and the informants prior to the study. No specific information about the interviewers’ were reported to the informants except their occupation. There was no presence of anyone else but the informants and the interviewing researchers during the interviews. The interviews were held in an open conversational style with support from the interview guide. The length of each interview was about 60 min. The research team discussed and analyzed the interviews continuously, two to three interviews at a time, throughout the data collection phase. This further dictated the recruitment of the next informant, i.e. strategic selection of informants was performed, which in turn continued until “saturation” in the data occurred. Memos about preliminary assumptions, ideas and theoretical reflections were written during the interviews and analysis process when indicated.

The simultaneous collection and analysis of data was performed between October 2020 and November 2021, by the first author (ES) and second author (CAA) with supervision by the third author (JB).

The interview recordings were de-identified and encoded, after which they were transcribed in verbatim. The names and birth dates of the interviewed informants were not used in this documentation. Transcripts were not returned to participants for comments. Data was reported only at group level. Although quotes from the interviews have been used as examples, no individual participant can be identified by outsiders. No repeat interviews were carried out.

### Data analysis

Qualitative content analysis is a well-described and systematic method that contains several steps. Central concepts in qualitative content analysis are meaning-bearing units, meaning condensation, coding, categorization, and themes. Meaningful units are the parts of the text that contains relevant and important information regarding the purpose and question of the study. Condensation of sentences involves a more manageable shortening of the meaning-bearing units, without losing important content. Coding means naming a condensed unit, and categorization means that several codes with similar content fall under one and the same category. During the analysis, a hierarchy often is noticed between the categories and therefore these can be divided into categories and subcategories. A theme is the latent more interpretive “red thread” that runs through the themes underlying categories [27]. The themes themselves can be seen as labels, which either consists of words the informants have said, or words formulated by the researchers [27].

The analysis of the data in this study began with a close reading of the transcribed interviews. Meaning-bearing units were identified and further condensed into shorter pieces and coded. The codes were then arranged into categories with underlying more descriptive subcategories, which was exemplified by quotes from the patient interviews.

The analysis and interpretation of data was initially inductive but towards the end of analysis, a deductive approach was used to achieve trustworthiness (credibility, dependability, and transferability) of the result [26]. This means that if a certain result pattern was noticed from the analysis of previous interviews, the researchers could ask specific questions to verify this pattern, in coming interviews. This by using a strategic but also theoretical recruitment of informants to test the saturation of the data and the prominent result theory. Saturation in data was first perceived after analysis of eight interviews, after which two more interviews were conducted. When no additional data were added and no change in the result picture emerged, saturation of data in the study was considered achieved. In the end of the analyze work a discussion between all participating researchers took place and several variants of theme structures were proposed and reworked until everyone was satisfied. The established study result was experienced, by all the researchers, to reproduce what the entire text from all the informants together contained, and this at different levels of abstraction. In other words, reliability in the collection and interpretation of data and the credibility in the interpretation of statements and arguments, was shown.

Important in the data collection and analysis process is the ability to adopt theoretical sensitivity and specificity within the research group. This includes reflection on one’s own pre-understanding, including not letting pre-understanding prevent the research process, but rather using it to identify and analyze important data [30]. The research group included two professional dentists in oro-facial pain and function (ES, CAA), an experienced dentist and qualitative researcher (JB), and a maxillofacial surgeon and senior consultant with long experience in treatment of HNC (GK).

### Ethics

The study was approved by the Swedish Ethical Review Authority, reference number 2021 − 01258. All respondents asked were willing to participate and gave both oral and written informed consent.

## Results

### Result picture

The result picture revealed three themes in connection to the informants’ experiences of affecting emotions, symptoms and use of pre- and post-treatment coping strategies, beginning with “*Management of simultaneously influencing mind-sets before cancer treatment*” and after treatment *“Experiences of becoming a pale shadow of oneself*” followed by “*Handling contextual influencing experiences after cancer treatment*”. This framework of themes formed a pattern with underlying categories and more descriptive subcategories (Tables 1, 2 and 3), as illustrated by quotations from the informants in the following text.

### Theme 1. Management of simultaneously influencing mindsets before HNC treatment

The identified categories of emotions related to the emotions experienced when receiving the cancer diagnosis and when preparing for the cancer treatment. The emotions of being “*Scared and worried*” and “*Lonely and disappointed*” reflected a more negative impact, while emotions of being “*Relieved and confident*” conveyed a more positive, relaxed and trusting attitude. The participants managed the aroused emotions by using the categories of coping strategies, ”*Applying a positive mind-set*,” ”*Searching for social support*,” and ”*Trusting the healthcare system*”.

To hold in mind - There may be interactions between experienced emotions, and coping strategies in the ongoing context, including additional impact factors such as other individuals.

**Table 2:** Management of simultaneously influencing mindsets before cancer treatment

Emotions		Coping strategies	
*Categories*	*Subcategories*	*Categories*	*Subcategories*
*Scared and worried*	- Feeling traumatized - Fearing the future - Feeling responsibility for others	*Applying a positive mindset*	- Being determined to survive - Staying positive and strong - Using positive self-talk
*Lonely and disappointed*	- Feeling let down - Feeling objectified - Lacking a holistic care approach	*Searching for support*	- Relying on family and friends - Needing professional support - Being cared for by others
*Relieved and confident*	- Feeling being helped - Feeling calm and secure - Feeling grateful to receive Swedish care	*Trusting the healthcare system*	- Relaxing from worrying - Being well informed - Relying on treatment methods

#### Scared and worried

Being diagnosed with a serious illness left the informants with feelings of *being traumatized* and shocked by the situation, and they reported experiences of *fear of future* and death upon receiving the cancer diagnosis. *“I was so shocked, I mean, what if I die? I wasn’t ready for that!”*

Subsequently the patients also foresaw the potential consequences of their deaths for their families and near ones. They reported senses of worry and *responsibility for others*, partly for the financial problems the disease or death could cause the families, but also for how the families would manage in life in general. This in turn caused emotions of anxiety and sadness, as one interviewee stated, *“How is my family going to manage if I die.”*

#### Lonely and disappointed

The interviewees who lacked support from both the professional caregivers and from their own social network *felt that the cancer care had let them down*, and they reported a deep feeling of being alone, as one interviewee communicated, *“I had no one to support me, I had to manage it all by myself.”* Another dimension of patient-reported loneliness was the sense of not being at the center of their own lives, and that other things or people were more important and superior to themselves, which also gave them a feeling of being alone and not embraced by others. Additionally, a lack of information about how to get in contact with the care providers diluted the feeling of being alone and left out, *“I didn’t know what to expect from the treatment and I felt like I never really knew who I could turn to, to get help or information.”*

While the interviewed patients reported an overall positive experience of the Swedish healthcare system when it came to information about the diagnosis, therapy planning, and prognosis, they also reported negative perceptions of the healthcare professionals in their treatments of the patients as individuals. The survivors mediated *feelings of being objectified* and not seen and heard as individuals with individual needs: *“I felt like a package that needed to go from this treatment to that treatment and sent from this place to that place, without anyone ever asking me once how I felt.”*

Further, the informants *lacked a holistic approach* in care, where the patient’s entire life situation including different influencing factors were considered: *“No one ever asked me what was going on in my life, and what I needed to be able to manage this.”*

#### Relieved and confident

The interviewed survivors shared that, despite the bad news upon receiving the cancer diagnosis, they also felt relieved. They had experienced that something was wrong and had previously sought help for symptoms such as a new lump or difficulty swallowing, without any results. The diagnosis gave them a confirmation of their concerns. This further gave the patients a *sense of being helped* and that they now could *get the care they needed*. As one interviewee recounted, *“I was so relieved that I got a diagnosis since I could perceive that something was wrong and now I could finally get help.”* They also reported experiences of *feeling calm and secure* because now they knew and no longer had to worry.

The participants experienced *gratefulness to receive Swedish care*, when they perceived the cancer care in Sweden as advanced: “*I am grateful that I got sick in this country where the healthcare system is so good.”* This made them more confident that they had a good chance of survival: *“I trusted the healthcare system here, to help me survive”.*

#### Applying a positive mind-set

Despite the fear of death, upon cancer diagnosis the majority of the interviewed patients had *a determination to survive.* They explained that they used this determination as a strategy to handle the fear and worry of what was ahead: *“I just felt I had to survive, so I decided that I will, because I must.”*

They expressed that they understood and felt the importance of the attitude towards the situation. If they *stayed positive and strong*, things would go well and their chances of survival would increase, as one interviewee stated, *“I will be strong, because attitude means everything, I’ll just manage thi*s.”

The *use of positive self-talk* as a coping strategy to handle the anxiety and worry about the diagnosis and treatment ahead was also reported. The informants convinced themself that everything would be all right and that they would survive by repeating it to themselves: *“I told myself every day to be strong, you can do this, nothing can come in your way if you just stay strong.”* Some of them also explained the self-talk strategy as not overthinking, to just ”*do the job*” and ”*get it done*”. This was a way to explain that they planned to just follow the instructions and treatments without too much thinking of possible consequences: *“I said to myself, keep going and you will be fine.”*

#### Searching for support

There was an overall strategy upon HNC diagnosis to search for support to stay positive and to manage the fear of the cancer. The survivors expressed that they *needed to rely on family members as well as friends and colleagues* to manage the stress and anxiety, as one informant said, *“I knew I had to rely on my husband.”*

They also expressed a need for supportive conversations at a higher level with, e.g., a psychotherapist: *“I wish I had a professional to talk to about how I felt.”* Finally, the overall feeling of *being cared for by others* was used as a strategy to keep up the fighting spirit: *“I was grateful I had my family as support. Without them I wouldn’t have made it.”*

#### Trusting the healthcare system

The informants explained that they used their trust in the healthcare system to calm their nerves and to *relax from worries*, stating for example, *“I know I’m getting the best care in the world, and therefore I am not worried.”* They felt confidence in leaving their lives in the hands of the healthcare professionals, and that they could be compliant to the treatment methods, because they *relied on them* to be well planned and resourceful: *”I had full confidence that they would do all they could to save me.”*

### Theme 2. Experiences of becoming a pale shadow of oneself

The informants report of negatively affecting symptoms and side effects after cancer treatment, presented in Table 3, were categorized into “physical, cognitive, and social impacts”.

To hold in mind -many vital functions could be affected as the symptoms could co-vary with each other and with anatomical locations of the HNC disease.

**Table 3:** Experiences of becoming a pale shadow of oneself

*Categories*	Physical affects	Cognitive affects	Social affects
	Pain Mucosal problem Chewing problem Reduced mouth opening ability Dysphagia Nutrition problem Loss of weight Mouth dryness Flavor change Odor impairment Hearing loss Voice change Malaise	Pain influenced Anxious Depressed Afraid Worried Slow Uninitiated Decrepit Circumstantial	Lonely Social avoidant Socially distant Socially isolated Unsupported Reduced working ability Deteriorated economy Hard to plan life

#### Physical impacts

The informants reported problems with *pain*, both during and after the cancer treatment: *“To get the treatment was painful in itself but the pain in my jaws is still there.”*

The pain mainly occurred in the head and neck area. The mouth was a frequent location, often together with *mucosal problems*, e.g., sores. Pain upon oral functions such as *chewing* and *mouth opening* was also reported. The pain could be long-lasting and sometimes reported as remaining several years after treatment: *“I still have problem when it comes to eating. I cannot open my mouth wide enough and chewing harder food hurts in my jaws.”*

Further, the participants reported problems with *dysphagia* during treatment and subsequent *problems with nutrition* and *loss of weight* after treatment. There were also reports of *mouth dryness* and *flavor changes* that could develop into chronic problems: *“It’s been over ten years and my mouth is still dry and food doesn’t taste the same.”*

Depending on the tumor localization, there were reports of *hearing loss* and *voice disorders*. Lastly, the informants reported general feelings of *malaise* during treatment and of feeling *week and tired* during and after treatment: *“During the treatment I felt like I was so weak that I was close to death all the time. Now I have this remaining tiredness that makes me feel like my entire body is slower than before.”*

#### Cognitive impacts

*Anxiety* and *depression* were reported as severe and sometimes long-lasting cognitive side-effects, both during and after treatment. The participants mediated feelings of being constantly afraid and worried and often the anxiety was related to recurrence of cancer and fear of death: *“I’m still worried that the cancer might reoccur, and what if I won’t make it this time?”*

The informants also conveyed that they felt *slower* and had *less inclination and energy to take initiative*, both in the short-term and the long-term perspective: *“It feels like my brain is slower now. I can’t make decisions like I used to.”* Another expression was that they felt like a weaker *decrepit* version of their former selves: *“I don’t feel like my former self, this is a sluggish version of who I used to be.”* This resulted in a feeling of personal decay where everything that needs to be done now felt *circumstantial* and cumbersome: *“I used to do what was ahead of me with enthusiasm, but now I feel like everything that needs to be done is a burden.”*

#### Social impacts

Feelings of *loneliness* were reported as a consequence of many parts of the treatment and the treatment outcome. The informants conveyed that due to various dysfunctional side effects, they avoided exposing themselves to other people and therefore *avoided social interaction*s even with family and close friends.

It could also happen that other people exposed dislikes towards the patients and further *distanced themselves* from them. The participants expressed a feeling of being *socially isolated*: *“It sometimes feels like even my kids avoid me nowadays, maybe because they find it difficult to see me like this.”*

Loneliness due to few social contacts and loss of family and relatives was emphasized, which could adversely affect the social assistance. In addition to this, factors such as *poor follow-up* and *poor support* from care were raised, whereby the patient’s vulnerability further increased. They could end up feeling alone in an enormous void, where the ability for self-handling was put to the test: *“I had no one to turn to, and I felt like I needed a support system, at least from the healthcare, but I was alone.”*

In addition, the informants gave reports of *reduced working ability* and consequently *deteriorating economy*, which could imply a burden of support of others in *a hard-to-plan life: ”I cannot work full-time anymore and of course this affects my economy.”*

### Theme 3. Handling contextual influencing experiences after cancer treatment

The identified emotional categories related to experiences after treatment, where “*Shocked and disappointed*” and “*Concerned and unsupported*” reflected a negative impact and “*Grateful and forward-looking*” revealed a more positive impact of treatment on the informants. The identified coping strategy categories related to the experiences of handling the impairments and discomfort of treatment symptoms by ”*Appreciating life*”, ”*Networking socially*”, and ”*Adapting to ‘the new life’*”. All interviewed patients had experienced severe side effects after treatment, and they had used different strategies, often in combination, to cope with them.

To hold in mind - There may be interactions between experienced emotions and symptoms, and further on with the use of coping strategies in the ongoing context.

**Table 4:** Handling contextual influencing experiences after cancer treatment

Emotions		Coping strategies	
*Categories*	*Subcategories*	*Categories*	*Subcategories*
*Shocked and disappointed*	- Feeling shocked over treatment outcome - Lacking supportive therapy - Lacking individualized aftercare	*Appreciating life*	- Greeting life - Enjoying and relaxing - Changing their perception on life
*Concerned and unsupported*	- Having difficulties adapting to physical and mental consequences - Feeling like a burden - Feeling unempathetically treated	*Networking socially*	- Obtaining social nutrition - Being socially active - Searching for support
*Grateful and forward-thinking*	- Trusting the treatment plan - Seeing the treatment outcome as affordable - Feeling curiosity about what tomorrow might bring	*Adapting to* *”the new life”*	- Preventing new problems from arising - Accepting own personality change - Being in control of own life

#### Shocked and disappointed

The informants mediated a sense of *feeling shocked over treatment outcomes*. They had not been able to foresee the consequences, that is how life would change after the cancer treatment. They reported an overall disappointment regarding the lack of help and support from caregivers. They experienced *lacking supportive therapy* upon cancer diagnosis, and they also missed and felt a need of supportive therapy during and after treatment. The *individualized information* about how harsh the treatment could be were experienced as very poor. In addition, they reported lacking information on treatment-related symptoms and of long-lasting side effects that still affected their lives. On top of this, they *experienced difficulties getting help and support*, including getting no information on how to contact the caregivers: *”I thought I was going to die, not from the cancer itself, but from the treatment I received to fight the cancer - I felt like the living dead”.*

Furthermore, the informants described a *lack of follow-up meetings and individualized rehabilitation plans.* Some conveyed that they had not received information about possibilities of rehabilitation: *“Is there no one else like me who wants to fight? Who wants to take my hand so we can fight together?”*

#### Concerned and unsupported

The physical and mental consequences from the cancer treatment were experienced as very *difficult to adapt to*. Some informants reported difficulties interacting with others due to their changed appearance, and problems working like they had previously done. Statements like, *“I prefer not to meet anyone at all, because my appearance is so changed”* and *“I don’t want to talk to anyone on the phone, they can hear that my teeth and half the face is gone”*, were frequent.

These consequences had a large social impact on their lives, with feelings of being constantly worried. They had lingering thoughts about the cancer, the possibility for it to come back, and a fear of death, and they perceived themselves as lonely and *a burden* to their families: *“I constantly feel like my symptoms are a burden for my loved ones.”*

A general need for help was expressed, both when it came to professional support from the healthcare system or as social support from family and friends. The informants sometimes felt that they were *treated unempathetically* by the caregivers, and they expressed feelings of not being seen or heard. At the same time, they noted a difference between the two care systems, where access to dental care was perceived as easier and the support more personal and individualized than the medical care: *“I could contact my oral surgeon at any time, and he would know how to help me. I never felt that support from anywhere else.”*

#### Grateful and forward-thinking

In contrary to the patients who felt disappointment over their cancer care, there were also patients who expressed a lot of gratefulness. They felt grateful for the treatment and support they had received and experienced the whole healthcare system as very professional. They reported that they had been positive to the treatment plans and that they *trusted in them*. They also experienced good support and good availability from the healthcare systems throughout the entire treatment and afterwards: *“I feel grateful for every day that I´m alive.”*

Even though the treatments were rough, and the symptoms and side effects were long-lasting, they expressed that they were grateful over the treatments and *saw the outcome as affordable*: *“Life goes on and I’ve put this behind me - I’ve sailed past all the obstacles.”*

Some of the interviewed patients also conveyed a sense of wanting to live on, as they could think ahead in life with *feelings of curiosity: “I am curious about what tomorrow might bring.”*

#### Appreciating life

Even though the symptoms and the remaining side effects both physically and mentally were described as severe and consuming, the patients expressed a lot of *gratefulness over being alive*: *“I appreciate every breath I take.”*

The informants also experienced a sense of not wanting their everyday difficulties to take over their highly valued everyday life by *enjoying and relaxing*. They especially valued the feeling of luck and happiness of being surrounded by family and friends, stating, *“I think so much more about my surroundings and my fellow human beings, and thank every day that we are here.”*

They also experienced that they had changed their perceptions of what was important in life, and that their whole *perspective of life had changed*: *“I look at life in a whole new way, none of the things that were important before are important anymore.”*

#### Networking socially

As patients who handled their worries and anxieties upon cancer diagnosis by obtaining social support, the informants reported that they used social networking to handle and cope with the consequences after cancer treatment. The informants felt impaired and strained as they relied on the nearness and concern from their families: *“The family had to help a lot, they are the biggest and most important support.”* The informants also reported that taking care of a pet could serve as nearness and by extension give *social nutrition*.

*By being socially active* and having multiple social contacts in different contexts, the patients felt that their conditions for adapting to everyday difficulties were improved. It also strengthened the patients’ ability to *take on challenges* and solve problems: *“I’m so grateful for my husband, I thought every day, and I hope that everyone who goes through what I do has that support.”*

#### Adapting to “the new life”

The interviewed patients expressed different types of strategies on how they learned to manage and cope with the consequences of their treatments. Striving to *prevent new problems from arising* and trying to prevent a worsening of symptoms were methods used.

An overall experience was that they had to accept the new person they have become, that is *their own personality change*:*”This is the new me and I have accepted it.”*

Furthermore, in order to adapt to the new normal life by learning new ways of functioning and doing things, it was found useful to have the feeling of *being in control of one’s own life*. After adapting and when they felt safe, supported, seen, and heard, the interviewed participants reported improved conditions for finding own new ways for adaptation.

## Discussion

This study aimed to explore HNC patients’ experiences and management of emotions and symptoms before, in connection to and after cancer treatment, and the care and support provided during this. The results from the interviewed patients’ expressions revealed a multidimensional picture of perceived affects and ways to handle them. For future implication, a strategy is suggested, to identify patients in short of the support they individually require, managing recovery, based on this study’s informants’ own experiences of the support they would have demanded.

### Emotions

The informants’ perceived emotions were often contradictory, containing both negative and positive thoughts, which they had to deal with simultaneously.

Patients whose mindset were characterized by agony and a lack of foothold seemed to be stuck in a shocked and disappointed mood and in continuous rumination about the disease, its multitude of consequences, and a sense of having been deserted with it all. A sense of abandonment has earlier been described in palliative and terminally ill patients and has also been described for HNC patients during the treatment phase [30, 31].

Some study participants experienced feelings of mistrust towards the healthcare system rather than a wish for additional contact. They expressed that they had not been properly attended to or listen to by the healthcare personnel, in a manner they described as unempathetic. Also, not been involved in the decision-making process or given the chance to understand the therapeutic opportunities. They described feelings of that the treatment was thrown at them and that they felt like a product, that just needed to pass the process. This resulted in a sense of insecurity towards the care given to them. To receive individualized care, to be listened to and trusted, and to feel secure, are mentioned in a study from 2009 as important for survivors [32].

At the same time, some patients pointed out that they absolutely believed that they could and would be saved by the healthcare personnel and trusted fully in them to defeat the cancer with the treatments they were prescribed. They felt seen and secure, and expressed a high satisfaction of the information, preparation and care given.

### Symptoms

The reported symptoms and difficulties after the cancer treatments were strikingly difficult, not least the cognitive side effects. These can result in personality changes and reduced or more limited internal resources for coping with difficulties, which should be taken into account in planning, rehabilitation and when giving support.

In terms of TMD, pain in the facial area has earlier been reported as the third most common persisting pain in individuals overall, and described as a risk to be engaged and sustained in a vicious circle, supported by fear and a counterproductive behavior [33]. Dysfunction in the facial area, which is the inevitable consequence of cancer treatment modalities in the facial area, could also be part of a similar vicious circle.

### Coping strategies

Strategies for coping, described in this study, seemed to differ regarding on whom they relied. When possible, the strategies emanated from own (sufficient) strength. With reduced access to this, they relied on (available) social support from family and friends. If social support was lacking, they depended on (arbitrary) support from the healthcare system.

Individuals are said to possess different coping capacities, which may also differ between the influence of perception and abilities within the respective individual or events in the surrounding context. Individual factors that influence capacity could be health and energy as well as social skills and beliefs about self-management and control. Environmental factors that have been described to impact coping capacity are, for instance, social support availability, material resources, and attitudes that needs to be confronted [34].

It has been earlier described that one way to endure difficulties is to try to find positive emotionsin a negative experience [17, 29]. Some informants seemed to be able to handle their fear of deaththrough a positive-mindset and a ”I must survive and therefore I will”-type of thinking. These individuals also expressed a strong ambition to fight against death during the treatment even though the side effects were rough, and the pain and suffering were overwhelming. They seemed to find meaning through the struggle, supported by their positive and forward-thinking strategy.

Other patients felt that they suffered from a series of consequences from their illness and treatment to which they had to surrender. They were no longer able to work as they used to because of the large number of socially limiting side effects and the worry about the financial issues arising from this affected them greatly. This was illuminated recently in a study pointing at HNC patients’ financial distress due to work disability [16]. They further expressed a need to have a short time perspective and to take each day as it came. A short time perspective have previously been described among HNC patients [30].

Apparent was, that some informants after treatment had reprioritized and re-evaluated most things in life and changed their perspectives of what is important. They reported a better understanding for other people, better relationships with near ones, and a loss of interest in material matters in life. This is in line with other studies which have reported similarly altered perspectives [16]. They mentioned the acceptance of the “new normal” as a way of continuing with life after cancer and in a way turning a death threat into a gain in life.

### Needs

The informants described their relation to health care in a multitude of ways, with different expectations, needs and wishes.

They expressed needs of individualized and more holistic care, where they would be properly and professionally attended and listen to by the caregivers. Further, to be given proper pre-treatment information and to be involved in the treatment therapy. Some individuals, even though they considered themselves adapted to life post-treatment, highlighted a need for the healthcare system to give better information and guidance concerning life post-treatment, to address the patients´ specific rehabilitation needs and opportunities. As well as support opportunities of how to manage symptoms and long-lasting side effects of the treatment. This in line with earlier studies [35–37].

### Care givers

It seems that the availability and access to counseling, both at the time of diagnosis, before treatment, and in the course of rehabilitation, have varied. It appears unclear for the informants where and how to get in contact with the healthcare when in need of support. Some have turned to the private healthcare sector for psychological care, and some have received it from their primary care facility. Thus, a clear, systematic pathway for the patients to receive counseling seem to be lacking. Additionally, support availability appears to be limited. This unclear pathway might be the consequence of HNC care in Region Västra Götaland being divided between several care sectors. Since these patients are treated by different departments, in tandem as well as sequentially, it becomes difficult to be certain which department is responsible for the patients’ mental well-being during and after treatment. The perception was that the dentalcare professionals such as oral surgeons were easier to reach when in need of support or discussion. Dental care, in this study, was also perceived to use a more person-centered approach to the care recipients.

### Adaptment to a new life

The assistance needed to adapt to life after cancer treatment seems to be an area that could benefit from further development. There is a need for the healthcare system to provide a much more clearly organized and well-informed support process. The healthcare system could improve in identifying the patients in need of support at an earlier stage and provide them with the right information and guidance when they need it. It is possible that if counseling was presented as an option for all patients in the beginning of the treatment, this could enhance the feeling of being seen as a whole person and increase the perception of the treatment as emphatic. In addition, it is naturally of great importance that the healthcare personnel possess both the knowledge and the experience to maintain a professional, empathic and supportive approach towards the patients.

### Future implications

The healthcare system thus has the potential capability to enhance patients’ coping prerequisites, through improved attention to individual patients’ attitudes and improved availability. Such efforts are also in line with the ontology behind person-centered care [38]. Since individual factors like control beliefs are described to influence coping capacity, it might also be possible to “screen” for individuals with an additional need of support from the healthcare system. Such a screening might be possible to implement in the HNC care program as a way of recognizing the patients in need of extra support on time. For example, strategies for the alleviation of pain and anxiety for those HNC patients who cannot independently generate enough of their own resources, will be very important factors to further explore, to improve quality of life in this burdened group of patients.

This study contained questions explicitly asked about emotions and coping strategies both before and after treatment. A tendency was shown, when reading thru the interviews, that patients who were able to enter their treatment with a positive mindset seemed to adapt more easily to persistent affects and to the inconveniences that was brought on them, after treatment. This could not be seen as an outcome or result in this qualitative study, but something that ought to be further explored, in future quantitative studies. An interesting question then could be - Can the availability of useful coping strategies before cancer treatment predict the management of post treatment symptoms and complaints?

### Strengths and limitations

The chosen approach provided a breadth of collected expressions on the aimed query, which provided a deeper picture of HNC patients’ experiences. The qualitative approach with interviews was well-suited for capturing patients’ experiences, thoughts, and day-to-day challenges during and after HNC treatment [39]. Use of a semi-structured interview guide ensured that certain topics were included in the discussions. However, the course of the interviews was still directed by the survivors, since it is considered easier to probe with a non-standardized approach in interviews [40, 41].

Content analysis was the base of the analysis method in this study, and the method’s condensing procedure provided a concrete basis for compilation of slightly diverging reports of similar expressions, while the determination of saturation provided effectiveness in recruitment and analysis [29, 42]. The addition of methods enabled interviewing and disturbing no more participants than necessary, and prevented the analysis from over-expanding, thus losing dependability through a shifted view of the content over time. Because analysis was conducted after every second or third interview, the possibility that the researchers’ pre-understanding of the previously collected information could color future data collection and analysis was reduced. The reliability of a study is high if another researcher easily can follow the decision made by the first researcher during the work. This was reached through constant discussion of the results within the research group, searching for agreement.

The theoretical selection towards the end of the study meant that the researchers could test for a certain pattern/theory if this was noted from the analysis of previous interviews. This was achieved through asking specific, more in-depth questions in the coming interviews. This could be seen as a way of validating a theme or category [43].

All recruitment and interviewing were performed in cooperation by the three first authors, and the analysis and writing of the manuscript involved all authors who had different occupational- (general dentist, orofacial pain specialists and HNC-cancer maxillofacial surgeon), scientific- (PhD student, PhD, associated professor, experienced qualitative researcher), and experience-related (age, sex, years in occupation, occupational focus) backgrounds, and thus, pre-understanding prerequisites.

Limitations of the study could be that the conclusions were built upon the participants’ experiences and the researchers’ interpretation, which requires theoretical sensitivity in the data collection and analysis process, within the research group. Further the study’s data were collected from the same medical and dental institutions in the Västra Götaland region of Sweden. Therefore, the results cannot be generalized to all HNC patients. Another limitation could be that the interviews took place at one time-point, hence we did not explore the changes of experiences over time within individuals. Furthermore, a sample size of 10 participants could be considered as small, but since a constant comparative analysis of the data was implemented, saturation of data occurred and directed the sample size.

## Conclusions

This study presents a broad description of HNC patients’ need of individualized care throughout their whole treatment as well as the supportive aftercare. The results of this study highlight that the need for care varies depending on individually reported symptoms as well as patients’ description of experienced emotions and strategies to cope with them. Patients demand a more holistic approach from the healthcare system, as there are other things in life that can affect the possibilities for management and relief of symptoms and side-effects. It is also evident that the HNC disease implicates a complicated physical area with a large set of healthcare specialists involved. The physical area of disease presentation makes the consequences of lack of support tangible and non-dismissive. Moreover, the number of specialists involved in treatment and care, and their separated organizations for instance within both medicine and dentistry, might be important factors contributing to lack of a cohesive care situation. However, there is a great opportunity to develop a more holistic care, if the care could include a multidisciplinary collaboration.

Improved care should address the individual patient’s need for support, in terms of symptom and lack of function, but also be grounded in his/her own capability and own support structure. A way forward through co-building a prosperous coping capability is suggested. This effort must include a clear pathway regarding post-treatment rehabilitation in all regards. It should involve an individual care coordinator with a thorough knowledge and understanding of the HNC patients’ treatment and the individual patients’ conditions. This coordinator should have the capacity and mandate to facilitate support within the wide range of potential specialists needed to enhance recovery and “acceptance of the new normal”, in order to maintain or improve quality of life. Perhaps this preferably is provided by the oncological care who have the main responsibility for the patients.

## Data Availability

The dataset generated and analyzed in this study are not publicly available due to the sensitive nature of the answers from the informants. Please contact the corresponding author for requests of data from this study,
